# Visual metaphors promote place bonding in urban marathon participation through emotional involvement and spatial meaning reconstruction: a ZMET–PLS-SEM study

**DOI:** 10.3389/fpsyg.2026.1921873

**Published:** 2026-08-26

**Authors:** Yuchen Wang, Hongcai Song, Jiangsu Lu, Jie Wei

**Affiliations:** School of Design, Jiangnan University, Wuxi, China

**Keywords:** emotional involvement, eventscape, place bonding, PLS-SEM, spatial meaning reconstruction, urban marathon participation, visual metaphor, ZMET

## Abstract

**Introduction:**

Urban marathon participation provides a real-world mass-participation and active leisure setting in which runners perceive environmental cues through continuous bodily movement and visual attention. However, how event-based environmental cues are transformed into emotional involvement, the psychological reconstruction of spatial meaning, and place bonding remains insufficiently explained. Taking the 2025 Wuxi Marathon as a case, this study examines the mechanism through which visual metaphors shape runners’ place bonding.

**Methods:**

A mixed-methods design was adopted. Study 1 used the Zaltman Metaphor Elicitation Technique (ZMET) with image-elicited interviews of 15 highly involved runners to identify visual metaphor cues and meaning-generation pathways. Study 2 translated the qualitative findings into a PLS-SEM model and tested it using 348 valid questionnaires.

**Results:**

Event brand image recognition, landscape–cultural aesthetics, scene transition perception, and order-and-care experience significantly affect event emotional involvement and the psychological reconstruction of urban spatial meaning, which in turn promote place bonding. Urban spatial meaning reconstruction has a stronger effect on place bonding than event emotional involvement, while order-and-care experience plays an important role in linking event participation with urban perception.

**Discussion:**

The findings suggest that visual metaphors in urban marathon participation can be understood as a mechanism of place meaning generation. They show how runners transform event-based environmental cues into emotional involvement, the psychological reconstruction of spatial meaning, and place bonding, revealing a place-based pathway related to supportive urban experience and psychological well-being.

## 1 Introduction

Environmental psychology research emphasizes that place-related responses arise from dynamic transactions between individuals and socio-physical environments. Through these transactions, environmental settings are perceived, interpreted, and endowed with emotional or identity-related meanings (Berze and Dúll, 2024). Urban marathons provide a mobile context for examining this process, as runners engage with the host city through route-based movement, visual attention, and event atmosphere (Ouyang et al., 2022). Sporting events hosted in cities have been regarded as media connecting urban development, public space governance, and sociocultural interaction (Smith, 2009; Lowes, 2018). Planned eventscapes create linkages among physical, symbolic, and social spaces and influence city image, collective identity, and city brand communication (McGillivray, 2019; Oshimi and Harada, 2019; Yamaguchi, 2025). Compared with earlier discussions on event structure, organizational processes, and management quality (Emery, 2002; Gwinner et al., 2009), recent studies have increasingly incorporated participant-centered dimensions, including embodiment and affective experience, eventscape perception, and behavioral intention (Yazıcı et al., 2017; Guo et al., 2024).

Among mass-participation sporting events, this study identifies a type of urban marathon that can be understood as an urban experience-oriented marathon (Xu and Dai, 2025). Such events are grounded in in-person mass participation and unfold through open urban spaces, with the host city serving as the primary medium of experience (Herstein and Berger, 2013; Richelieu, 2018). Their routes connect roads, waterfronts, parks, cultural landmarks, campuses, service nodes, and public spaces into a continuous running encounter. In this process, runners perceive the city through bodily movement, visual attention, emotional fluctuation, and spatial transition. These marathons are characterized by strong city embeddedness, route-based visual exposure, and the integration of sport participation with place-based perception. This feature distinguishes them from trail marathons, stadium-based sport events, and online or virtual races (Sjukriana et al., 2025). Urban experience-oriented marathons therefore provide a distinctive setting in which the city functions simultaneously as the route of participation and the object of perception. This dual role makes them especially suitable for examining how event experience becomes urban meaning.

Recent research has gradually outlined an analytical trajectory from supply-side event provision to participant-centered experience in urban experience-oriented marathons. From the perspective of host-city attributes, urban functions and governance can significantly affect participants’ perceptions of event brands (Lin et al., 2024). From the perspective of the event experience process, factors such as eventscape cognition, participant roles, and motivations may further influence behavioral intention and value co-creation through mediating mechanisms such as affective identification, place attachment, and perceived value (Song et al., 2025; Zhang and Feng, 2026). Existing quantitative studies tend to treat event and city as corresponding variable dimensions and then examine the structural relationships between them. A smaller body of qualitative research has begun to explore how bodily practice, sensory experience, and rhythm perception during running events are associated with sense of place (Vink and Varró, 2021). Nevertheless, insufficient attention has been paid to how runners psychologically organize the environmental, spatial, and social cues encountered during marathon participation and how these cues are transformed into emotional involvement, spatial cognition, and place-based responses. Clarifying this process is necessary for explaining how temporary and mobile encounters with the city acquire the affective and interpretive significance needed to support place bonding. This mechanism also lacks systematic quantitative validation. In fact, the city image formed in such events emerges through the interaction among body, route, and place (Bale, 2004). Within this process, the role of sensory mechanisms, especially visual perception, in urban marathon experience remains underexplored. Studies on city branding and image communication have shown that vision is an important medium through which spatial experience is identified, organized, and communicated (Zhang et al., 2025). Existing research has also indicated that visual perception plays a dominant role among sensory triggers in tourism landscape experience (Buzova et al., 2021). In the context of urban experience-oriented marathons, however, visual experience is more processual and situated. Runners’ visual perception cannot be reduced to the passive viewing of environments or event symbols. It is a process in which specific visual cues are selected, remembered, and transformed into meaning during long-distance movement, bodily fatigue, emotional fluctuation, and scene transition.

In response to these issues, this study takes “visual metaphor” as an analytical lens for connecting bodily experience, environmental perception, and place-based affect. The concept is used here to denote a cognitive mechanism through which runners understand, organize, and express marathon experience and urban perception through concrete visual images and their contextual associations. This study addresses three research questions. First, in urban marathon participation, which urban visual scenes, spatial cues, or cultural images are repeatedly remembered by runners across the complete event experience and organized into a shared visual metaphor system? Second, how are these visual experiences transformed into higher-level event meanings and urban meanings, and how do they further affect runners’ emotional involvement and spatial cognition? Third, how do the cognitive and affective mechanisms triggered by event-based visual metaphors further contribute to relatively stable and sustainable place bonding?

To answer these questions, this study adopts A mixed-methods design that combines qualitative and quantitative approaches. First, the Zaltman Metaphor Elicitation Technique (ZMET) is used to identify the relational structure linking visual attributes, experiential consequences, and place-based values. This process generates a mental map that connects participants’ visual metaphors with place bonding and provides the basis for developing the antecedent constructs of the model. Second, partial least squares structural equation modeling (PLS-SEM) is used to quantitatively examine the validity of the proposed framework.

This study takes the Wuxi Marathon, held in Wuxi, a Jiangnan city in China, as its empirical case. The Wuxi Marathon is one of the most well-known marathons in China and was awarded the World Athletics Gold Label in 2023. Its route design is characterized by distinctive seasonal features and place-specific landscape encounters, making it a suitable case for this study. By integrating the qualitative generation mechanism of visual metaphors with the quantitative validation of a structural model, this research explains how the visibility of the city in urban experience-oriented marathons is further transformed into intelligibility and place bonding.

The contribution of this study lies in the construction of a visual-metaphor-driven framework for place meaning generation. It reveals how visual experience, spatial organization, and social interaction jointly shape place bonding in urban marathon participation. This framework provides a new explanatory pathway for understanding how short-term endurance sport and active leisure participation can become more enduring place-based psychological relationships. It also offers empirical evidence for improving runners’ emotional experience, route perception, and place-related meaning-making. The strategic implications further suggest that event visual design, route organization, and visible service support can enhance the inclusiveness, adaptability, and affective resilience of urban public experience.

## 2 Theoretical background

### 2.1 Visual metaphor in urban environmental meaning-making

The physical urban environment provides perceivable visual cues through material interfaces such as urban forms, landscapes, signage, and spatial configurations. In research, these resources can be understood through the concepts of urban image and urban representation, while metaphor offers a cognitive and interpretive lens. Urban image concerns how a place is perceived; representation addresses how urban meaning is organized and presented; and metaphor explains how subjects move from environmental perception to meaning generation. Lynch’s theory of urban imageability, proposed in 1960, emphasizes that a place can form a clear and stable mental image in the observer’s mind through its own distinctive qualities (Lynch, 1964). As research has paid increasing attention to the connections between place image, cultural meaning, social imagination, and identity orientation, the city has also been understood as a representational system that produces meaning through symbolic and material resources (Aiello, 2021a). From the perspective of meaning representation, the key concern is how urban images are organized into a coherent imagination through media, narratives, and visual-material resources. Through this process, the visible city becomes a communicative structure. Because the meanings conveyed through images are often tacit and difficult to verbalize directly, metaphor provides an important analytical perspective for examining their underlying cognitive structures. Metaphor-elicitation methods further offer a pathway for accessing these latent meanings.

In urban studies, metaphor has moved beyond linguistic and rhetorical description and has been understood as a mode of thought, often defined as understanding and experiencing one thing in terms of another. Earlier studies used metaphorical descriptions of urban concepts to explain urban complexity or policy communication (Nientied, 2016). Recent research further emphasizes that conceptual metaphors can organize complex urban phenomena and provide context-sensitive frames for interpreting urban processes (Rogers, 2023; Luger and Schwarze, 2024). At the visual level, metaphorical meaning arises from the conceptual relations that viewers derive from images in context, enabling concrete visual forms to evoke broader experiential and cultural meanings that are difficult to articulate directly. Such meanings do not reside in isolated visual elements but emerge through the relationship between what is depicted and the concepts it brings into association (Scott, 1994; Refaie, 2003; Forceville, 2024). The interpretation of urban images is also shaped by viewers’ accumulated social and spatial trajectories, which influence how urban space is cognitively organized and represented (Dias and Ramadier, 2015).

On this basis, the present study adopts visual metaphor as an analytical lens for examining how runners use concrete images and contextual associations to organize their understanding of the event and host city. In urban marathon participation, this meaning-making process is embedded in movement along the route and in runners’ changing bodily relations with the surrounding environment. The qualitative phase identifies the visual cues and relational structures through which these interpretations are expressed.

### 2.2 Embodied and situated cognition in mobile urban environments

Influenced by the mobility turn in human geography and embodied approaches in cognitive science and environmental psychology, scholarship across urban, tourism, and leisure studies has increasingly examined spatial experience through bodily movement and interaction with the environment. Mobilities scholarship understands movement as a socially meaningful practice shaped through its routes, rhythms, and contexts (Sheller and Urry, 2006; Cresswell, 2010). Research on embodied cognition further suggests that cognitive activity is grounded in sensorimotor systems and develops through the body’s engagement with its environment (Barsalou, 2008). These perspectives provide a basis for understanding urban marathon participation as a mobile form of experiencing and interpreting the city. Marathon experience also unfolds through the continuing adjustment between runners’ bodily rhythms and the rhythms of the event and urban environment (Edensor and Larsen, 2018).

Runners experience the eventscape through bodily states that change throughout the race. As physical effort accumulates, attention may shift, allowing the same environmental setting or visual-material cue to acquire different experiential salience at different stages. Recent studies of participatory sport events have begun to conceptualize embodied experience as a relational process formed through bodily engagement with material settings and affective atmospheres (Larsen, 2019; Xu and Dai, 2025). Embodied cognition helps explain how changing bodily states shape perception and the incorporation of environmental conditions into personal experience and memory. Situated cognition addresses another aspect of this process by highlighting how the meaning of environmental cues is shaped by the immediate context of action. The significance of an eventscape cue may vary according to the stage of the race, the runner’s ongoing activity, and the surrounding environment (Wei and Wang, 2018). Urban settings and event-related cues may consequently acquire different experiential meanings as participation unfolds.

Continuous movement may also influence how dispersed urban locations are integrated into spatial understanding and how event-related cues become associated with particular route segments. Research on spatial learning suggests that direct navigation supports the sequential acquisition of environmental knowledge and may facilitate the integration of places encountered at different moments (Ishikawa and Montello, 2006). Active exploration can further influence the development of cognitive maps (Chrastil and Warren, 2012; Brunec et al., 2023). A marathon route brings urban locations that may otherwise be experienced separately into a continuous spatial sequence. Event cues embedded along this sequence may also become associated with particular stages, settings, and forms of participation. Taken together, these theoretical perspectives provide a basis for considering how movement along a marathon route may influence runners’ interpretation of the eventscape, perception of spatial relations, and understanding of the host city. The specific form and extent of this process remain open to empirical investigation.

### 2.3 From event experience to place bonding

Person–place relationships may develop when environmental experiences acquire personal significance and become incorporated into how individuals understand and remember a place. Place attachment research conceptualizes such relationships as involving affective, cognitive, and behavioral processes through which people connect with particular settings (Scannell and Gifford, 2010). Research has also distinguished between place meanings arising through direct perception and action and place interpretations that develop more gradually through reflection (Raymond et al., 2017). In an urban marathon, the host city becomes part of a demanding and personally significant experience. Impressions formed during the event may therefore remain salient afterward and continue to be elaborated through memory and interpretation. Related sport-event research also shows that perceptions of an event and its destination are associated with place attachment and subsequent behavioral intentions, while participation in running events may shape how individuals experience and relate to the host city (Kaplanidou et al., 2012; Vink and Varró, 2021). Together, these findings suggest that event participation can provide an initial context for the formation of person–place relationships.

Place bonding is well suited to conceptualizing this developing relationship because it emphasizes the multidimensional ties formed through meaningful interactions between activities and settings (Hammitt et al., 2006). Although place bonding and place attachment overlap conceptually, place bonding gives particular attention to how person–place relationships emerge and deepen through time, use, and continued experience (Hammitt et al., 2009). Such bonds may begin during an initial encounter and do not necessarily require long-term residence or extensive prior familiarity with a place (Cheng and Kuo, 2015). This developmental emphasis is especially relevant to urban experience-oriented marathons, where intensive bodily involvement and the personal significance of participation may initiate a place-based relationship within a limited period. Place bonding therefore captures both the initial formation and developmental character of this relationship.

In existing event research, however, the affective, cognitive, and behavioral dimensions of place-related responses are often examined through separate constructs (Song et al., 2025; Zhang and Feng, 2026). This separation is useful for identifying specific pathways, but it provides less insight into how these dimensions converge within a broader person–place relationship. Place bonding therefore provides an integrative framework for examining how perceptual, affective, and interpretive meanings generated through event participation are organized within such a relationship. The remaining theoretical question is how a short-term event experience acquires sufficient emotional and interpretive significance to support the formation of a relationship that may persist beyond the event.

## 3 Research design

**Figure 1:**
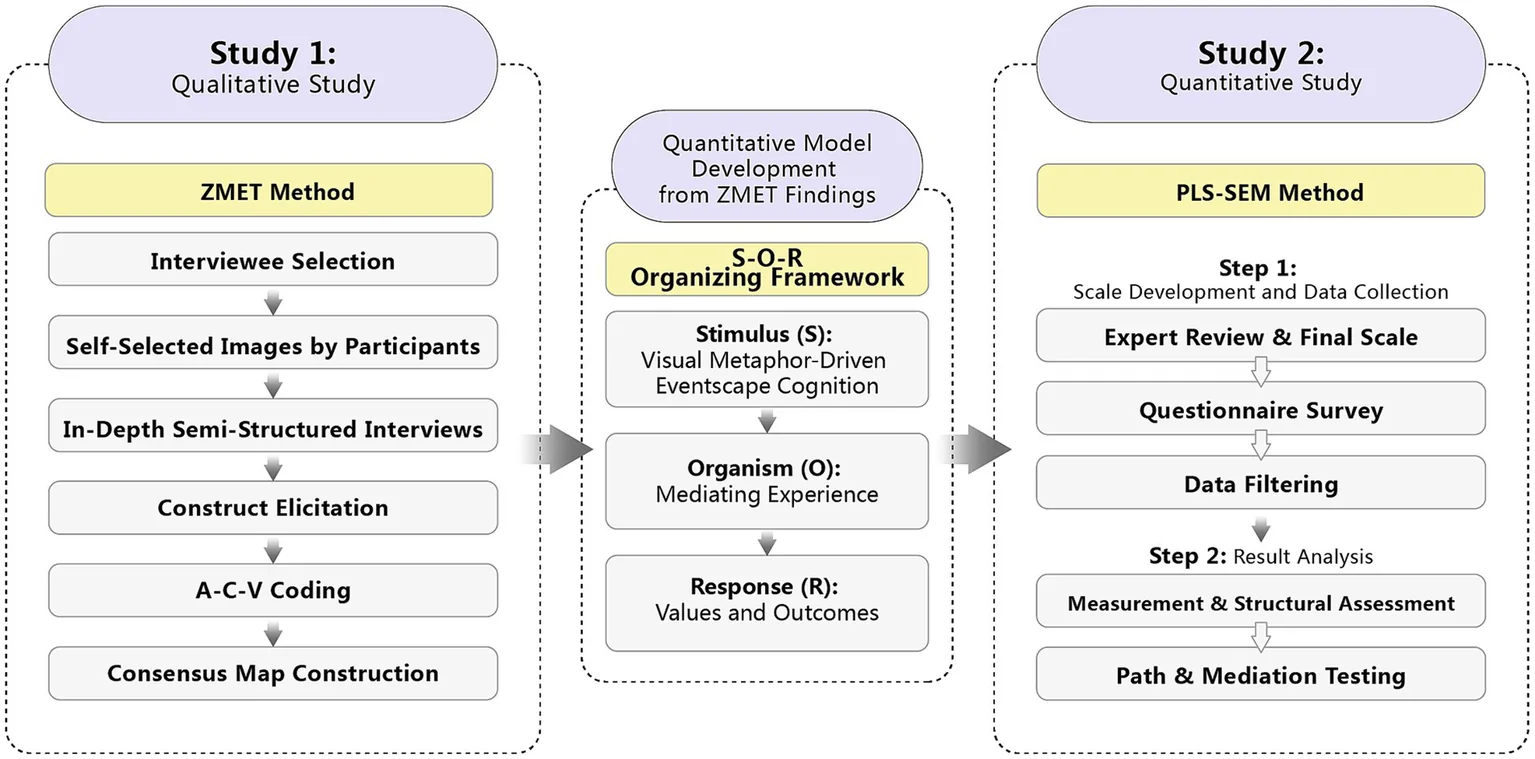
Research roadmap.

As shown in Figure 1, this study adopts an exploratory sequential mixed methods design, in which qualitative findings provide the basis for the subsequent development and quantitative testing of contextually grounded constructs (Creswell et al., 2003; Bakhsh et al., 2024). This design is appropriate because the visual-metaphorical meanings associated with urban marathon participation are situated, visually mediated, and closely connected to participants’ lived experiences of the event. The research procedure consists of three linked stages.

The first stage, Study 1, focuses on visual metaphor elicitation and construct generation. Using ZMET, we conducted in-depth interviews with highly involved runners in an urban marathon and constructed a consensus mental map based on the attribute–consequence–value logic chain of runners’ eventscape experiences. The second stage focuses on construct transformation and serves as the point of integration between the qualitative and quantitative phases. The recurrent constructs and meaning chains identified through ZMET were operationalized for quantitative measurement, while the S-O-R framework informed the organization of eventscape perceptions, internal experiential responses, and place-related outcomes. Thus, the qualitative findings shaped both construct operationalization and structural model specification. The third stage, Study 2, focuses on structural model testing. Using partial least squares structural equation modeling (PLS-SEM), we examined how the eventscape cognition dimensions identified in Study 1 contribute to place bonding through event emotional involvement and urban spatial meaning reconstruction. This sequence links qualitative meaning elicitation and construct development to quantitative testing of the proposed mechanisms.

All empirical work in this study centers on the Wuxi Marathon held in March 2025. Its route integrates Jiangnan waterscapes, traditional cultural settings, modern urban spaces, and a distinctive university campus segment into a continuous running experience. The event is typically held during Wuxi’s cherry blossom season, and cherry blossom imagery serves as a prominent element of its visual identity. These characteristics make the event suitable for examining how runners interpret the visual and spatial dimensions of the eventscape through continuous route-based participation.

## 4 Study 1: visual metaphor elicitation in a mobile urban environment

Study 1 employed the Zaltman Metaphor Elicitation Technique (ZMET) to examine how runners organize visual-material cues and place-related meanings across the event experience. Metaphor-oriented image methods generally follow two pathways: identifying metaphors in existing textual, visual, or multimodal materials (Forceville, 2002; Schmitt, 2005; Group P, 2007), and eliciting participants’ latent meanings through image association and projective interviewing (Zaltman and Coulter, 1995; MacKay and Couldwell, 2004; Matteucci, 2013). ZMET belongs to the latter pathway. By combining self-selected images with guided comparison and construct elicitation, it can access embodied, affective, and image-based experiences that may be difficult to articulate through direct questioning and organize them into comparable mental structures (Zaltman, 1997; Christensen and Olson, 2002).

Zaltman Metaphor Elicitation Technique has been applied in tourism research to examine destination image, symbolic communication, travel experience, and tourist values (Kong et al., 2018; Yoo et al., 2022; Xiang et al., 2023; Yin, 2023; Ma and Li, 2025). These applications support its suitability for investigating how place-related meanings emerge from visually mediated experience. Accordingly, Study 1 used ZMET to identify the recurrent visual cues, experiential consequences, and place-related values through which runners interpreted their participation in the Wuxi Marathon.

### 4.1 Methods

Recruitment for the ZMET interviews began immediately after the Wuxi Marathon on March 23, 2025. Through online runner communities, event-related social media platforms, and offline snowball referrals, the researchers invited runners who had participated in the event and were willing to take part in in-depth interviews to enter the initial screening process. According to Zaltman’s analysis, a small number of high-quality in-depth interviews in ZMET research can usually cover the major constructs in a consensus map (Xiang et al., 2023). Recent empirical studies using ZMET have commonly adopted sample sizes of 8–16 respondents (Cheng et al., 2025). Therefore, this study did not pursue a large sample size. Instead, it emphasized participants’ event involvement, richness of experience, and an appropriate balance in sample structure. Prospective participants were first invited to complete the Personal Involvement Inventory (PII) (Zaichkowsky, 1985), through which runners with relatively high involvement and willingness to articulate their experiences were selected.

In controlling the sample structure, this study considered gender, age and familiarity with Wuxi. According to official event statistics, the male-to-female ratio of participants in the 2025 Wuxi Marathon was close to 3:1, and non-local runners substantially outnumbered local runners. Finally, 15 formal interviewees were selected based on gender, age, and degree of familiarity with the city, as shown in Table 1.

**Table 1:** Respondents’ information.

Respondents	Gender	Age	Place of residence
P1	Male	42	Local
P2	Male	29	Non-local
P3	Female	31	Non-local
P4	Male	25	Non-local
P5	Male	34	Non-local
P6	Male	30	Local
P7	Female	36	Non-local
P8	Female	34	Non-local
P9	Male	37	Local
P10	Male	40	Non-local
P11	Male	45	Non-local
P12	Female	33	Non-local
P13	Male	31	Local
P14	Female	43	Local
P15	Male	35	Non-local

Formal interviews were conducted between March and April 2025. Seven days before each interview, after being informed of the research topic and interview procedure, participants were asked to recall the most impressive scenes from the complete event process, including pre-race city promotion, material collection, the race itself, and post-race experiences. They were then asked to independently select 8 to 12 scene images that could represent their strong event memories. These images could include official event photographs, photographs taken by companions, or other relevant images saved by the participants after the race. However, all images had to be selected by the participants themselves, and they had to be able to explain the relationship between each image and their own event participation. In this study, these images served as interview stimuli to activate participants’ memories of visual perception, emotional changes, and place meanings associated with the event.

Before each interview, the researchers again explained the research purpose, data use, anonymity principles, and recording requirements, and obtained informed consent from the participants. The formal interviews were semi-structured and proceeded sequentially around the images provided by each participant. The procedure was as follows (Thomas et al., 2025; Marinao-Artigas et al., 2026):

1. Visual narratives: Participants described, one by one, the event scene associated with each image, the reason for selecting it, their bodily state, and their emotional responses. Based on the research objectives, the interviewer flexibly raised follow-up questions to guide participants in further describing the visual attributes, experiential consequences, and deeper values associated with these scenes.
2. Description of missing images: Participants were asked whether there were any important “missing scenes” that could not be represented by the existing images. They then supplemented the content and meaning of these scenes.
3. Sorting: Participants classified the images according to their own understanding and explained the commonalities within each category, as well as the relationship between each category and their event participation.
4. Image comparison and construct probing: The researchers selected several groups of images from each participant’s materials and guided the participant to compare similarities and differences in terms of scene, atmosphere, bodily perception, city impression, and affective meaning.
5. Representative images: Participants were asked to select the image that best represented their Wuxi Marathon participation in order to further clarify the boundaries of the core constructs.
6. Expanded framing: Participants were invited to imagine what additional scenes might appear if the camera were pulled back from the representative image, and to describe the meanings of these imagined scenes.
7. Sensory association and metaphor extension: Participants supplemented the images with non-visual perceptions, such as sound, color, touch, spatial rhythm, and bodily state, in order to explain how visual scenes were connected with emotion and place-based understanding.
8. Summary and confirmation: Participants summarized their complete city-based encounter before, during, and after the race, and reconfirmed the important concepts extracted during the interview. After processing and summarizing all materials, the researchers checked whether the collected data contained the essential information and relational structures required for subsequent analysis.

The interviews were conducted through a combination of online and offline formats. Each interview lasted approximately 60 to 120 min, was audio-recorded in full, and was transcribed verbatim. Subsequent analysis was based on participants’ images, interview transcripts, and researchers’ notes. Following the ZMET procedure, the researchers identified consensus constructs and developed a visual metaphor consensus map. To improve coding reliability, two researchers independently conducted preliminary coding of the interview transcripts. After comparing their coding results, they discussed disagreements in construct classification. For constructs that were difficult to determine, the researchers returned to the original interview context and the participants’ explanations of the images for further verification until agreement was reached.

### 4.2 Results

The researchers extracted and classified concepts from the interview materials. Open coding was conducted on the 15 formal interview transcripts, and concepts with similar themes and semantic meanings were merged. This process identified 116 original constructs. A further examination of the number of newly added constructs by interview order showed that the first four respondents contributed 94 constructs, accounting for 81.0% of all original constructs. The first seven respondents contributed 108 constructs in total, accounting for 93.1% of the total. From the eighth respondent onward, the number of newly added constructs decreased markedly. Subsequent additions mainly involved marginal supplementary constructs and further refinement or verification of existing constructs. After the thirteenth respondent, only a very small number of additional attribute constructs emerged, and the fourteenth and fifteenth respondents generated no new original constructs. These results indicate that, at the current level of coding granularity, the conceptual structure of this study had reached a stable stage of saturation. This judgment is consistent with the practice in existing ZMET studies, which identify construct consistency and saturation according to the declining trend of newly added constructs. Following the attribute–consequence–value logic of ZMET, the researchers classified the constructs according to the distinction among external stimulus cues, subjective cognitive elements, and meaning- or value-related elements. To avoid confusion with the outcome variables in the subsequent structural model, this study refers to the three types of constructs in the consensus map as initial constructs, linking constructs, and terminal constructs. The 116 original constructs were ultimately classified into 60 initial constructs, 36 linking constructs, and 20 terminal constructs.

**Figure 2:**
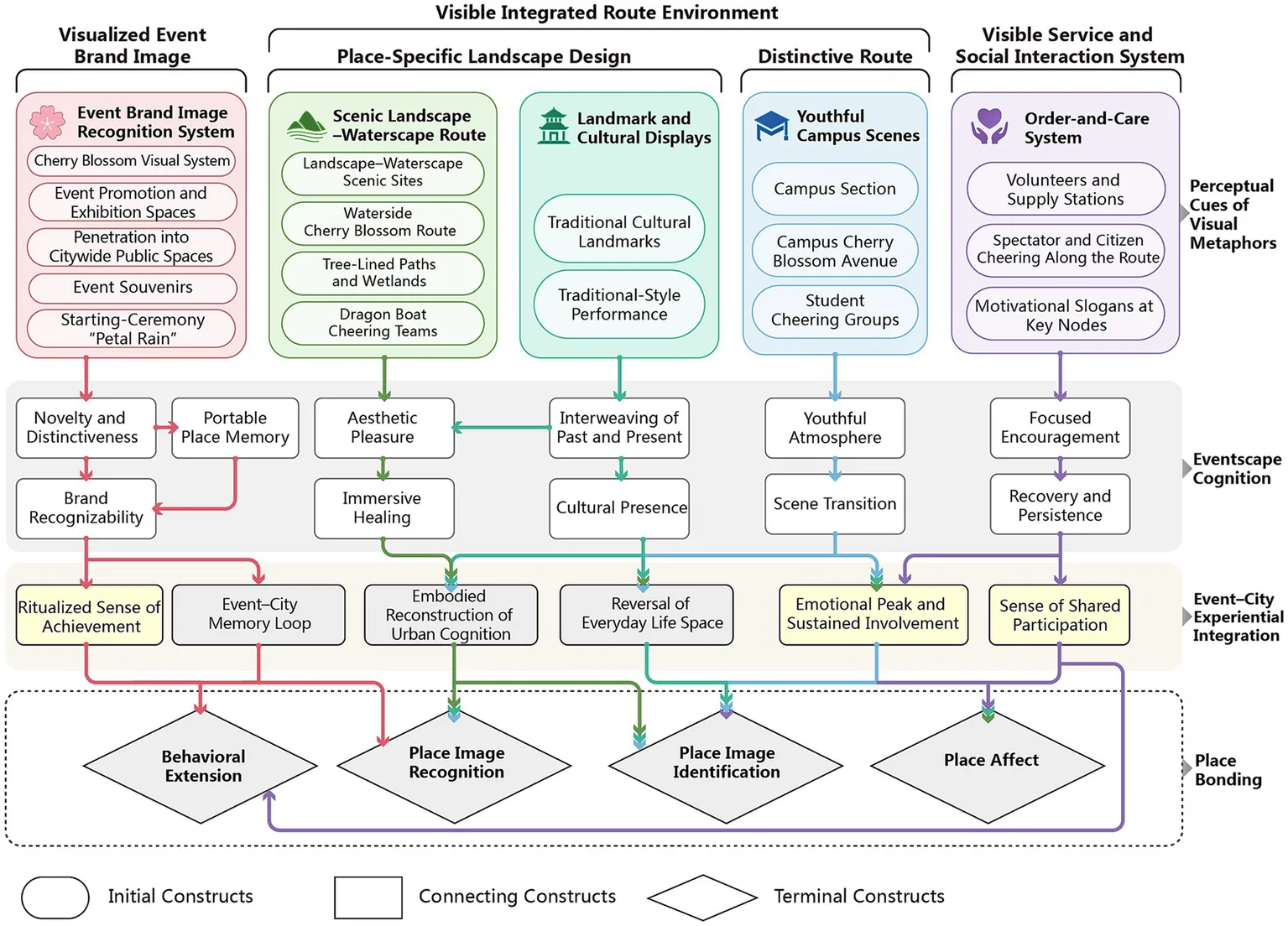
Consensus mental map.

The researchers further extracted consensus concepts and analyzed the relationships among them in order to develop the experiential mental map. According to the concept-convergence principle of ZMET, a consensus construct should be mentioned by at least one-third of the respondents, and a relationship between constructs should be mentioned by at least one-quarter of the respondents to be included in the consensus map (Zaltman and Coulter, 1995). Based on this principle, 17 initial constructs, 17 linking constructs, and 4 terminal constructs were retained, forming the consensus mental map shown in Figure 2. Building on the attribute–consequence–value meaning chain of ZMET, this study further refined the metaphor chains. The attribute layer, corresponding to the initial constructs, refers to highly perceived visual metaphor cues in the context of urban experience-oriented marathons. The consequence layer, corresponding to the linking constructs, connects attributes with values and represents runners’ cognitive and experiential integration of the eventscape and the event-city relationship. The final value layer, corresponding to the terminal constructs, points to comprehensive place meaning and place bonding.

As shown in Figure 2, across runners’ complete pre-race, in-race, and post-race encounters with the Wuxi Marathon, the highly perceived visual metaphor cues mainly fall into five categories: the event brand image recognition system, the scenic landscape–waterscape route, landmark and cultural displays, youthful campus scenes, and the order-and-care system. Representative examples of metaphor elicitation are presented in Table 2. The mental map further reveals four pathways through which the visual metaphor system of the Wuxi Marathon generates meaning.

**Table 2:** Representative examples of metaphor elicitation.

**Respondent/** **Photo No.**	**Interview materials**	**Photo**	**Attributes**–**consequences**–**values**
P14-08	The medal and finisher souvenirs were novel, distinctive, and strongly place-specific. Even the local food, xiaolongbao (soup dumplings), was designed in pink. The materials were consistent with the overall event design and served as good event souvenirs. After bringing this gift home, seeing it immediately reminded me of the event and the place. These souvenirs turned cherry blossoms into a city symbol of Wuxi, left me with a strong impression of this beautiful city, and made me look forward to returning.	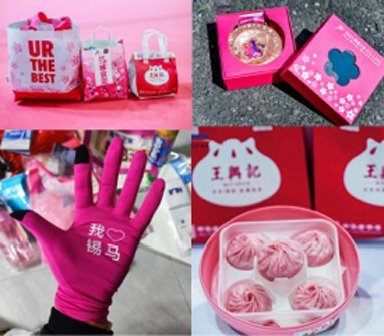	Event souvenirs → Novelty and distinctiveness → Portable place memory → Brand recognizability → Event–City memory loop → Place image recognition + Behavioral extension
P6-07	The 8–12 km section was rich in variation, with transitions among broad avenues, small bridges, and lakeside routes. The continuous waterscape, lake views, and specially designed cherry blossom path formed a highly aesthetically pleasing section, which matched the event slogan of “running in a painting.” It relieved my fatigue. I felt that the route organized the most place-specific landscapes together, allowing me to become immersed in the romantic atmosphere of the city and to experience its distinctive Jiangnan waterside character.	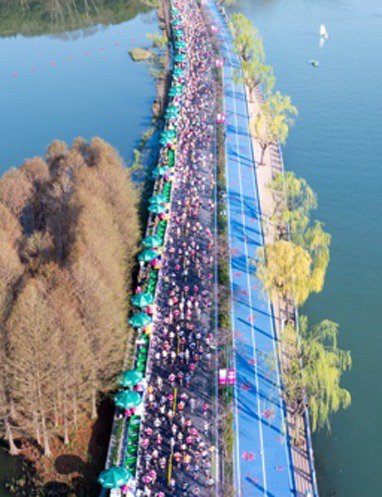	Landscape–Waterscape scenic sites + Waterside cherry blossom route → Aesthetic pleasure → Immersive healing → Embodied reconstruction of urban cognition → Place image recognition
P3-02	This archway is a well-known historic building in the area. Running under it gave me a sense of moving between history and modernity, and I could feel the atmosphere of traditional culture. This road is usually only open to cars, but this time I could run through it, which felt completely different from everyday experience and very open. This immersive encounter allowed me to truly experience the city’s historic and cultural charm, and I began to like this place.	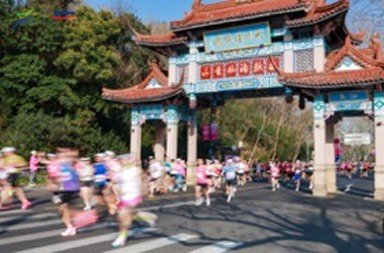	Traditional cultural landmark → Interweaving of past and present → Cultural presence → Reversal of everyday life space → Place image identification + Place affect
P2-11	Entering the campus section, I immediately felt a youthful atmosphere. The sudden change in the surrounding environment lifted my emotions to a peak and gave me renewed motivation to continue. It felt as if my distant university years had briefly returned, while also allowing me to sense and appreciate the youthful vitality of Wuxi more deeply.	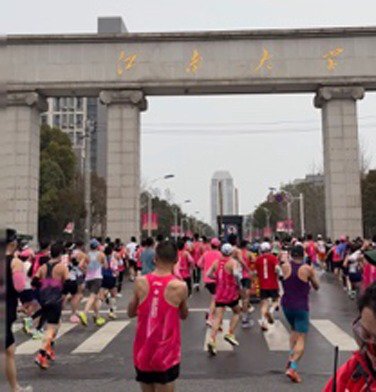	Campus section → Youthful atmosphere → Scene transition → Emotional peak and sustained involvement → Place image identification + Place affect
P8-10	In the second half of the race, a striking slogan reading “break through the wall” was posted on an underpass. This stage coincides with the marathon “wall,” when glycogen depletion can cause a sudden drop in performance. The slogan immediately encouraged me to keep going. The underpass and archway also created a spatial sense of “passing through,” making the idea of breakthrough more direct and embodied. This detail made me feel the care embedded in the place.	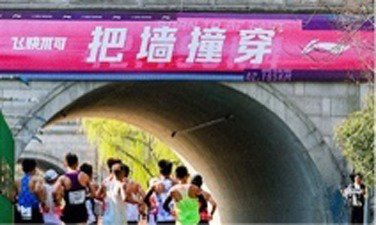	Motivational slogan at a key node → Focused encouragement → Recovery and persistence → Emotional peak and sustained involvement → Place affect

First, event visual identity constituted the most direct symbolic visual entry point for runners, leading to place image recognition and behavioral extension. The event brand system of the Wuxi Marathon takes cherry blossoms, a seasonal local feature, as its central theme. The color and graphics of pink blossoms are integrated into material design, the petal ceremony at the starting point, and environmental symbols. During the event, this distinctive visual identity permeated exhibition spaces, event settings, and public spaces across the city. These elements generated a sense of novelty and distinctiveness and strengthened runners’ recognition of the Wuxi Marathon brand. More importantly, these symbols did not remain at the level of event signage. They were transformed by runners into portable ritual memories, forming a memory loop from the event to the city. This process further promoted recognition of the city image and influenced future behavioral intentions.

Second, the route landscape system formed by the scenic landscape–waterscape route and landmark cultural displays directed runners toward embodied place image recognition and identification through sensory encounters that exceeded everyday life. Through route organization, the Wuxi Marathon transformed local waterfront resources and cultural landscape resources into a continuous event encounter. The natural landscape elements that generated strong perceptions included landscape–waterscape scenic sites, tree-lined paths and wetlands, the waterside cherry blossom route, and dragon boat cheering teams along the lakeside route. Landmark cultural elements included well-known traditional buildings and traditional-style performances along the route. Through long-distance bodily movement, runners continuously perceived Wuxi’s natural landscapes, Jiangnan character, and cultural symbols. Sustained emotional investment allowed them to experience an urban rhythm different from that of everyday life. The marathon enabled runners to move immersively through urban routes that they would be unable to walk through or perceive continuously in daily life. Within the embodied cognition of this landscape system, runners experienced aesthetic pleasure and immersive restoration. In this cultural atmosphere where the past and present were interwoven, they formed a reconstructed cognition of the city image. In this sense, participants reassembled their impressions of the city through bodily movement, visual continuity, and spatial transition, thereby forming place image recognition and affective identification.

Third, the distinctive campus route functioned as another important scene transition node beyond the landscape system, further evoking participants’ place affect and image identification. Passing through the campus is one of the recognizable route-node designs of the Wuxi Marathon. This scene was composed of the overall campus atmosphere, the campus cherry blossom avenue, and student cheering groups. These elements commonly generated heightened emotions among runners. They became important triggers for scene-rhythm transition and for the perception of the city’s youthful character, and they also contributed to more enduring place affect. During the race, the differentiated route progression from landscape and cultural landmarks to the campus space moved the route encounter beyond homogeneous road perception and created a more layered spatial rhythm.

Together, the second and third pathways indicate that urban experience-oriented marathons display and reorganize urban space, enabling runners to renew their understanding of city structure and place character. The route reorganized dispersed urban fragments, such as roads, bridges, lakeshores, tree-lined areas, and campus nodes, into a continuous embodied encounter. Through immersive running, runners formed a psychologically renewed mental map that exceeded conventional everyday experience.

Fourth, the visible service and social interaction system transformed event organization into affective support, place affect, identification, and willingness to approach the city again. Order-and-care cues included volunteers and supply stations, spectator and citizen cheering, interactive scenes, and motivational slogans at key nodes. These elements were also associated with the event brand system through volunteers’ uniforms, cherry-blossom-shaped sponges, and consistent pink slogans. They enabled runners to feel continuous support and focused encouragement during fatigue and psychological fluctuation, generating emotional involvement and shared participation among runners, citizens, and the city. Through visible order, care, and social interaction, the service system became a perception of urban warmth and encouraged future engagement with the city.

The interview data further show that the visual metaphor system identified in this case had a clear degree of group consensus. Although local and non-local runners differed in their specific interpretive emphases, they showed broadly similar perceptual pathways. Non-local runners tended to take cherry blossoms, lakeshores, landmarks, and Jiangnan culture as entry points for understanding Wuxi, emphasizing the novelty, city recognition, and tourism imagination generated by the event. Local runners were more likely to be moved by the reorganization of familiar spaces. They interpreted the transformation of distinctive roads, campuses, bridges, and lakeshores during the race as a form of urban rediscovery through a different mode of bodily engagement. Overall, runners’ perceptual processes followed a shared pathway: from systematic visual cues, through situated cognition and experiential evaluation, toward place image recognition and identification, place affect, and behavioral extension. This cross-group consensus also indicates that the visual metaphor system in urban marathons has a certain degree of inclusive explanatory power. It provides non-local runners with an entry point for recognizing the city, while also offering local runners an experiential framework for rediscovering everyday spaces.

### 4.3 Discussion

Overall, the visual metaphor system identified in this case was a meaning-generating structure formed by the integrated design of the route environment, together with the event brand image and social interactions embedded in the eventscape. Based on the semantic content of the consequence-layer constructs in the consensus map, Study 1 further divided the consequence layer of “event-city experiential integration” into two types. In Figure 2, the yellow blocks indicate participants’ affective evaluations at the level of individual psychology, whereas the gray blocks indicate experiential evaluations generated through the interaction between participants and urban space. The small-group ZMET findings show that, among the perceptual cues of visual metaphors, event brand image and social interaction elements were strongly associated with runners’ individual affective evaluations. They influenced place bonding through event recognition and socio-emotional support. By contrast, the integrated environmental design formed by the spatial interweaving of natural landscapes, cultural landmarks, and distinctive nodes primarily contributed to runners’ multi-layered, embodied, and non-routine psychological reconstruction of urban spatial meaning, which further generated terminal constructs such as place image recognition and identification. These findings provide the basis for the quantitative validation in Study 2.

## 5 Study 2: validation of the metaphor–place bonding model

To test the structural relationships identified in Study 1 with a larger sample, Study 2 developed a quantitative model based on the qualitative findings. Study 2 used the Stimulus–Organism–Response (S-O-R) framework to organize the construct relationships identified in the consensus mental map and develop a testable quantitative model.

### 5.1 Model source, construction, and hypotheses

S-O-R theory has been widely used in quantitative studies of environmental psychology, tourism experience, and destination image. It explains how external environmental stimuli influence attitudes or behaviors through internal cognitive and affective processing (Mehrabian and Russell, 1974; Jacoby, 2002). Existing studies often treat destination attributes, servicescapes, or cultural displays as the stimulus layer (Khairani et al., 2025); cognitive image, affective image, satisfaction, or affective identification as the organism layer (Eroglu et al., 2001; Yang et al., 2022); and revisit intention, recommendation intention, loyalty, or place attachment as the response layer (Zhu et al., 2025). In this study, the S-O-R framework was used to organize the “visual cues-situated cognition-experiential evaluation-place bonding” chain identified in Study 1 into a testable process structure. Initial ZMET constructs and earlier linking constructs form the stimulus layer, conceptualized as visual-metaphor-driven eventscape cognition. Later linking constructs form the organism layer, representing mediating experience. Place bonding forms the response layer.

**Figure 3:**
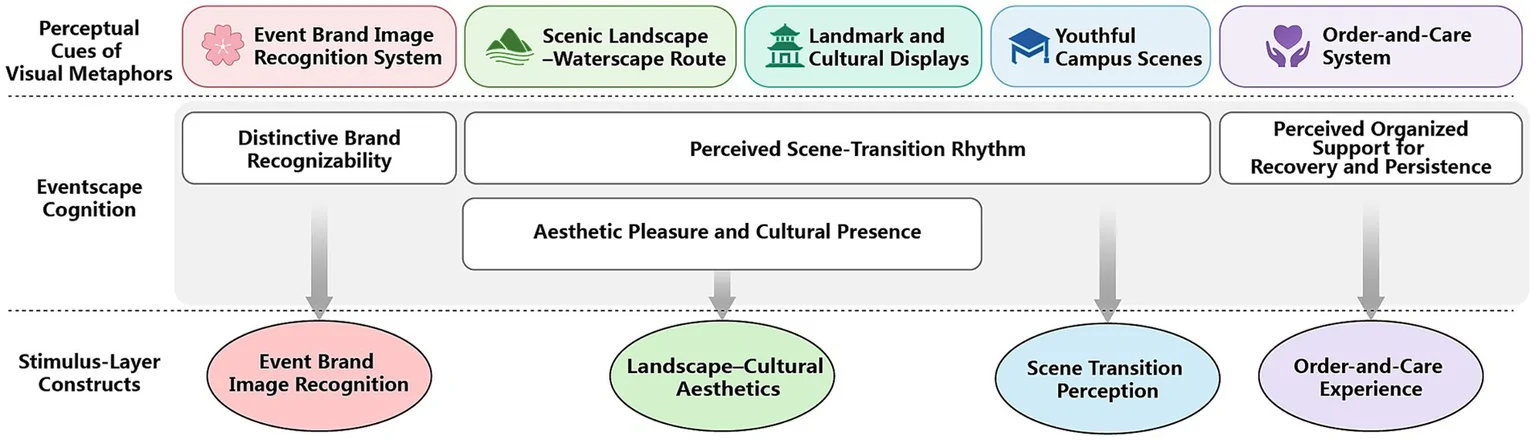
Derivation of stimulus-layer constructs from ZMET findings.

At the stimulus layer, this study extracted four antecedent constructs: event brand image recognition, landscape–cultural aesthetics, scene transition perception, and order-and-care experience. These constructs were abstracted from the four meaning-generation pathways identified in Study 1 (Figure 3). Event brand image recognition reflects runners’ recognition of the cherry blossom visual system, event colors, materials, ritual installations, and brand elements in urban public spaces. Landscape–cultural aesthetics reflects aesthetic judgment of route-based landscapes and cultural displays. Scene transition perception captures spatial rhythm and scene shifts across natural, cultural, campus, and road-space settings. Order-and-care experience derives from visible service and social support cues, including volunteers, supply stations, spectators, citizen cheering, and motivational slogans.

The organism layer includes two mediating constructs: event-based emotional involvement and urban spatial meaning reconstruction. Event emotional involvement was derived from the yellow blocks in the “experiential integration” layer of the mental map shown in Figure 2. It emphasizes runners’ immediate psychological engagement, peak experiences, ritual sense, and sense of community during the event. It reflects how visual-material cues are transformed into emotional involvement during event participation. Urban spatial meaning reconstruction was derived from the gray blocks in the experiential integration layer of the consensus map. In this study, it refers to runners’ psychological reconstruction of urban spatial meaning through route-based movement, visual perception, and event experience. It emphasizes how runners reinterpret, reconnect, and experience urban space through the event, thereby forming a new mental map of the city and a renewed sense of place.

**Figure 4:**
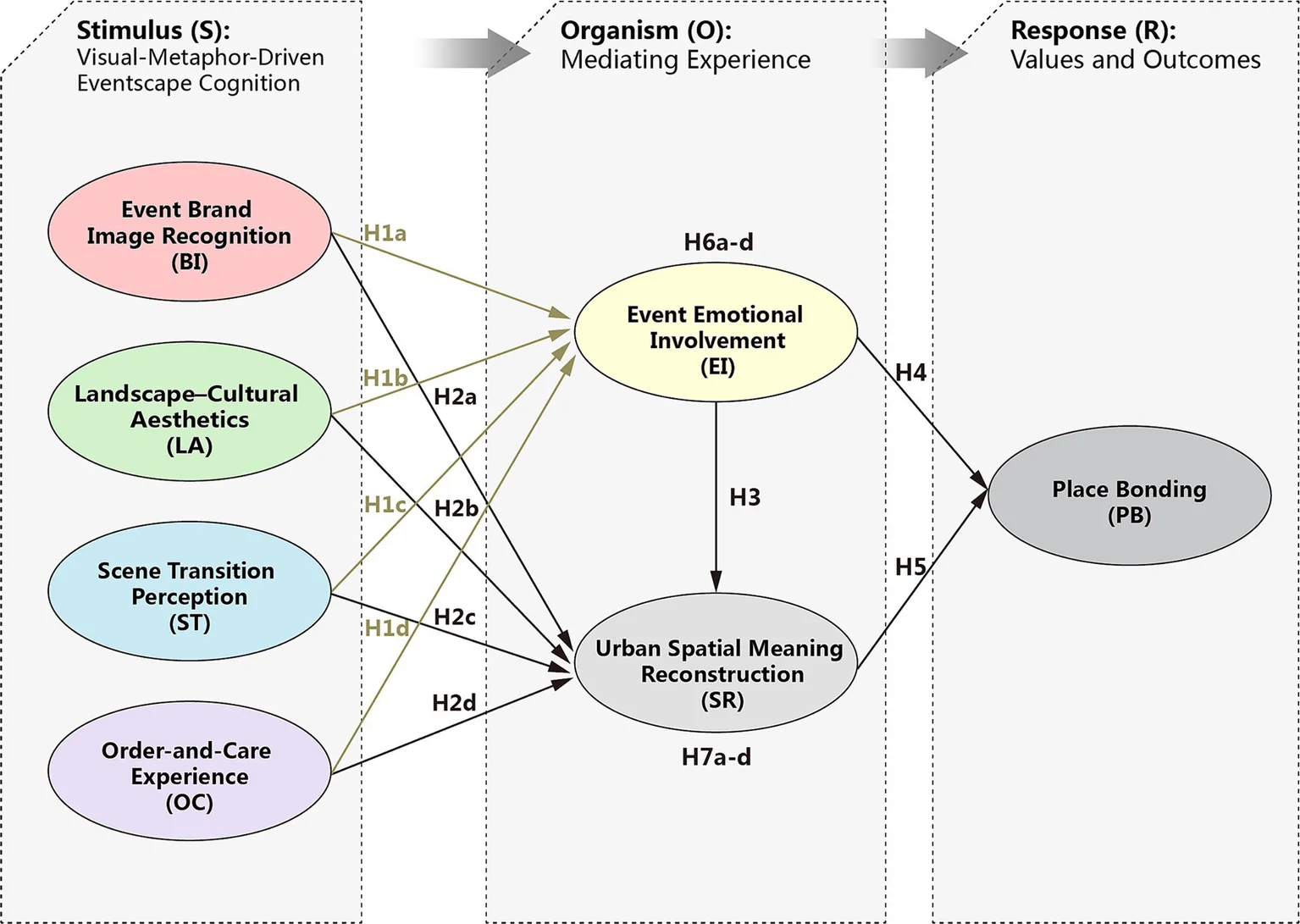
Research model.

In the response layer, place bonding captures runners’ post-event relationship with the host city, including place image recognition, place image identification, place affect, and behavioral extension. The complete model structure is shown in Figure 4. Building on the construct relationships identified in Study 1, the hypotheses were developed by combining the S-O-R process structure with targeted perspectives from brand experience and city image, environmental aesthetics, embodied and situated cognition, spatial learning, eventscape, social support, and person–place research.

The four dimensions of visual-metaphor-driven eventscape cognition may contribute to event emotional involvement through different mechanisms. Event brand image recognition may provide a coherent symbolic frame that enhances the emotional salience and memorability of participation (Brakus et al., 2009; Lin et al., 2024). Landscape–cultural aesthetics may generate positive environmental appraisal and experiential pleasure through the integration of scenic and cultural qualities (Kirillova et al., 2014; Buzova et al., 2021). Scene transitions may renew attention and produce variations in affective intensity as runners adjust their bodily rhythms to changing route environments (Edensor and Larsen, 2018). Order-and-care cues may render organizational support and social encouragement visible, thereby strengthening perceived support and shared participation (Lee et al., 2018; Carneiro et al., 2019). Accordingly, the following hypotheses are proposed:

*H1*: Visual-metaphor-driven eventscape cognition positively affects event emotional involvement.

*H1a*: Event brand image recognition positively affects event emotional involvement.

*H1b*: Landscape–cultural aesthetics positively affects event emotional involvement.

*H1c*: Scene transition perception positively affects event emotional involvement.

*H1d*: Order-and-care experience positively affects event emotional involvement.

Beyond their affective consequences, the four dimensions of visual-metaphor-driven eventscape cognition may also contribute to urban spatial meaning reconstruction through different interpretive and spatial processes. Event brand image recognition may link locally embedded event symbols to the city image, offering a recognizable frame for interpreting urban characteristics (Lin et al., 2024; Zhang et al., 2025). Landscape–cultural aesthetics may increase the perceptual salience of local environmental and cultural qualities and help runners organize individual scenes into a broader impression of the city (Kirillova et al., 2014; Buzova et al., 2021). Scene transition perception may support spatial meaning reconstruction by linking dispersed urban locations within a continuous movement sequence, thereby helping runners form a more integrated representation of the city (Ishikawa and Montello, 2006; Brunec et al., 2023). Order-and-care cues encountered at specific route locations may become part of runners’ judgments of the city’s orderliness, friendliness, and supportive qualities, because visible service performance can contribute to image formation (Lee et al., 2018; Lin et al., 2024). Accordingly, the following hypotheses are proposed:

*H2*: Visual-metaphor-driven eventscape cognition positively affects urban spatial meaning reconstruction.

*H2a*: Event brand image recognition positively affects urban spatial meaning reconstruction.

*H2b*: Landscape–cultural aesthetics positively affects urban spatial meaning reconstruction.

*H2c*: Scene transition perception positively affects urban spatial meaning reconstruction.

*H2d*: Order-and-care experience positively affects urban spatial meaning reconstruction.

Event emotional involvement may promote urban spatial meaning reconstruction because emotionally significant experiences are more likely to remain salient during subsequent reflection. Emotional peaks, ritualized achievement, and shared participation may give particular route scenes and interactions greater personal significance, allowing them to be integrated into a renewed interpretation of the host city (Raymond et al., 2017; Vink and Varró, 2021). Accordingly, the following hypothesis is proposed:

*H3*: Event emotional involvement positively affects urban spatial meaning reconstruction.

Event emotional involvement and urban spatial meaning reconstruction may promote place bonding through complementary pathways. Emotional involvement may give participation personal significance and affective salience, thereby strengthening emotional closeness to the host city and supporting subsequent intentions to revisit or re-engage with it (Scannell and Gifford, 2010; Kaplanidou et al., 2012). Urban spatial meaning reconstruction may operate through an interpretive pathway: by reconnecting route locations and assigning new meanings to ordinary or unfamiliar urban spaces, runners may form a clearer and more coherent representation of the city, which can support a developing place bond (Hammitt et al., 2009; Cheng and Kuo, 2015). Accordingly, the following hypotheses are proposed:

*H4*: Event emotional involvement positively affects place bonding.

*H5*: Urban spatial meaning reconstruction positively affects place bonding.

Taken together, the preceding relationships suggest two complementary mediating mechanisms. Event emotional involvement may transmit the affective significance of eventscape cognition to place bonding, whereas urban spatial meaning reconstruction may integrate dispersed environmental and event-related cues into a coherent understanding of the host city. These mechanisms are consistent with the S-O-R process structure, in which environmental perceptions influence place-related outcomes through internal affective and cognitive processing. Accordingly, the following mediation hypotheses are proposed:

*H6*: Event emotional involvement mediates the relationship between visual-metaphor-driven eventscape cognition and place bonding.

*H6a*: Event emotional involvement mediates the relationship between event brand image recognition and place bonding.

*H6b*: Event emotional involvement mediates the relationship between landscape–cultural aesthetics and place bonding.

*H6c*: Event emotional involvement mediates the relationship between scene transition perception and place bonding.

*H6d*: Event emotional involvement mediates the relationship between order-and-care experience and place bonding.

*H7*: Urban spatial meaning reconstruction mediates the relationship between visual-metaphor-driven eventscape cognition and place bonding.

*H7a*: Urban spatial meaning reconstruction mediates the relationship between event brand image recognition and place bonding.

*H7b*: Urban spatial meaning reconstruction mediates the relationship between landscape–cultural aesthetics and place bonding.

*H7c*: Urban spatial meaning reconstruction mediates the relationship between scene transition perception and place bonding.

*H7d*: Urban spatial meaning reconstruction mediates the relationship between order-and-care experience and place bonding.

The proposed relationships also imply a sequential mediating mechanism. Visual-metaphor-driven eventscape cognition may first heighten emotional involvement, which may increase the personal significance of event experiences and facilitate their subsequent integration into a renewed understanding of urban space. This process may ultimately strengthen place bonding. Accordingly, the following hypothesis is proposed:

*H8*: Event emotional involvement and urban spatial meaning reconstruction sequentially mediate the relationship between visual-metaphor-driven eventscape cognition and place bonding.

### 5.2 Survey design

This study used a structured questionnaire to collect quantitative data from runners who participated in the 2025 Wuxi Marathon. To ensure comparable event participation, respondents had to be at least 18 years old, have participated in the event, and be able to clearly recall route scenes, visual elements, and participation processes. A non-probability purposive sampling approach was adopted, and the questionnaire was distributed through multiple online runner communities and event-related social media platforms, with additional participants recruited through snowball referrals. The questionnaire items were mainly developed from the ZMET consensus map in Study 1 and were contextually adapted from established scales in tourism experience, destination image, sporting event experience, servicescape, and place bonding. All items were measured on a seven-point Likert scale, where 1 indicated “strongly disagree” and 7 indicated “strongly agree.”

Before the formal survey, three experts in city image research and two experts in sport management reviewed the questionnaire. A small-scale pretest assessed item comprehensibility and semantic clarity. Several items were revised based on expert feedback and pretest results. The final items are shown in Table 3.

**Table 3:** Measurement items of the research model.

**Variables**	**Items**	**Reference**
Event brand image recognition	The overall visual image of the Wuxi Marathon, with strong regional characteristics, left a vivid impression on me.	ZMET Interview; (Keller, 1993; Brakus et al., 2009)
	The prominent visual symbols of this event made it easy for me to recognize its brand characteristics.	
	Throughout the marathon, the visual elements across event promotion, start and finish installations, route design, and urban public spaces showed a high degree of consistency.	
	I clearly perceived the continuous presence of the event brand image throughout my participation.	
Landscape–cultural aesthetics	The distinctive regional landscapes along the route, including tree-lined paths, wetlands, waterside scenery, and cultural landmarks, made me feel pleased.	ZMET Interview; (Kirillova et al., 2014; Buzova et al., 2021)
	The inclusion of urban landmarks, scenic route sections, and cultural performances enhanced my aesthetic perception.	
	I clearly perceived the integration of waterscape landscapes, cultural landmarks, and urban culture.	
	The landscapes and cultural displays along the route jointly shaped the distinctive place-based aesthetics of this event.	
Scene transition perception	The transitions between different route scenes during the race left a strong impression on me.	ZMET Interview; (Geus et al., 2016)
	Changes in natural and cultural landscapes and road-space forms along the route made me clearly perceive shifts in event setting and rhythm.	
	Entering distinctive scenes, such as the campus section, created clear atmospheric transitions in the event.	
	The continuous transitions among different spaces and atmospheres made the event more layered and rhythmic.	
Order-and-care experience	The stage-based motivational signs and node settings along the route made me clearly feel that the race process was well organized and progressively guided.	ZMET Interview; (Du et al., 2015)
	The visible configuration of volunteers, supply stations, and service nodes made me directly perceive the orderliness and attentiveness of the event route.	
	The visible presentation of cheering, interactive scenes, and collective atmosphere along the route made me clearly feel supported and accompanied.	
	Visible order arrangements and care cues in the event environment gave me an overall impression that the race was thoughtful and well organized.	
Event emotional involvement	During the race, I developed strong emotional involvement in the event itself.	ZMET Interview; (Geus et al., 2016)
	This event gave me a clear ritualized sense of achievement.	
	I experienced strong emotional peaks in certain route sections.	
	During the race, I clearly felt integrated into a shared atmosphere of participation.	
Urban spatial meaning reconstruction	After completing the Wuxi Marathon, I developed a new understanding of the city’s spatial layout.	ZMET Interview; (Hillman et al., 2021)
	The race route helped me reconnect dispersed urban fragments that are not usually experienced together.	
	Some roads or spaces that are usually ordinary or difficult to run through outside the race context took on new meanings during the event.	
	This event made Wuxi’s urban space clearer and more memorable in my mind.	
Place bonding	This event helped me form a vivid and holistic impression of Wuxi.	ZMET Interview; (Hammitt et al., 2009)
	This event made me feel that I had established a certain lasting connection with this city.	
	I formed a positive and lasting favorable impression of this city.	
	I identify with the urban character and cultural image that this city presented through this event.	
	This participation strengthened my willingness to re-engage with this city in the future.	
	To me, Wuxi is no longer merely the host city of the event, but a city worth returning to and engaging with again.	

The formal questionnaire included screening questions, demographic information, and measurement items. The first page explained the research purpose, anonymity principles, data use, and voluntary participation. Submission was regarded as informed consent. The study protocol was approved by the Ethics Committee of Jiangnan University (JNU202509RB031).

Data were analyzed using SmartPLS 4. PLS-SEM was selected because the model was derived from ZMET findings and was suitable for theoretical extension and prediction-oriented analysis (Romo-González et al., 2018). The analysis included data cleaning, Harman’s single-factor test, measurement model assessment, structural model assessment, and bootstrap tests of direct, mediating, and sequential mediating effects, and supplementary robustness checks involving observed control variables, two-sided 1% winsorization, and an alternative bootstrap specification.

### 5.3 Results

#### 5.3.1 Data description

Study 2 collected 400 questionnaires, of which 348 were valid, yielding an effective response rate of 87.00%. As presented in Table 4, the sample included 192 male respondents (55.17%) and 156 female respondents (44.83%). It was mainly composed of young and middle-aged runners, with those aged 18–39 accounting for 75.00%. Respondents also had relatively high education levels and diverse occupational backgrounds. In terms of familiarity with Wuxi, 70.98% reported being “relatively familiar” or “very familiar.” The sample showed strong repeated participation: 92.24% had participated in the event at least twice, and 82.47% had participated in three or more sport events in the past year. Overall, the sample had sufficient event experience and city perception for model testing.

**Table 4:** Sample characteristics.

Variable	Category	Frequency (*N*)	Percentage (%)
Gender	Male	192	55.17
	Female	156	44.83
Age	18 ~ 24	55	15.8
	25 ~ 29	83	23.85
	30 ~ 39	123	35.34
	40 ~ 49	69	19.83
	50 and above	18	5.17
Education level	Below high school	15	4.31
	High school	35	10.06
	Associate degree	107	30.75
	Bachelor’s degree	149	42.82
	Master’s Degree or above	42	12.07
Occupation	Student	55	15.8
	Research staff	38	10.92
	Enterprise employee	132	37.93
	Government / Public institution staff	37	10.63
	Self-employed / Freelancer	41	11.78
	Worker / Service industry practitioner	29	8.33
	Others	16	4.6
Familiarity with Wuxi	Very unfamiliar: first visit	29	8.33
	General familiarity: visited 1–2 times before	72	20.69
	Relatively familiar: visited 3–5 times before	130	37.36
	Very familiar: local resident	117	33.62
Number of Wuxi Marathon participations	First time	27	7.76
	Second time	73	20.98
	Third time	138	39.66
	Fourth time or more	110	31.61
Frequency of sport event participation in the past year	About 1–2 times per year	61	17.53
	About 3–5 times per year	156	44.83
	About 6–10 times per year	106	30.46
	More than 11 times per year	25	7.18
Total		348	100

#### 5.3.2 Data analysis

1 Common method bias

Because the data were collected using a single questionnaire, common method bias may be a concern. Following commonly used statistical diagnostic procedures in previous studies, Harman’s single-factor test was conducted (Podsakoff et al., 2003; Fuller et al., 2016). As shown in Table 5, the first unrotated factor explained 37.103% of the variance, which is below the commonly used threshold of 40%. After rotation, the explained variance was distributed across multiple factors, and no single factor dominated. Therefore, the data did not show evidence of serious common method bias and were considered suitable for subsequent model testing.

2 Measurement model assessment

**Table 5:** Total variance explained.

Component	Initial eigenvalues			Extraction sums of squared loadings			Rotation sums of squared loadings		
	Total	% of Variance	Cumulative %	Total	% of Variance	Cumulative %	Total	% of Variance	Cumulative %
1	11.131	37.103	37.103	11.131	37.103	37.103	4.23	14.099	14.099
2	2.334	7.779	44.882	2.334	7.779	44.882	3.093	10.31	24.41
3	1.937	6.457	51.339	1.937	6.457	51.339	2.895	9.65	34.059
4	1.742	5.807	57.146	1.742	5.807	57.146	2.861	9.537	43.597
5	1.625	5.418	62.564	1.625	5.418	62.564	2.807	9.355	52.952
6	1.382	4.608	67.172	1.382	4.608	67.172	2.756	9.188	62.14
7	1.246	4.153	71.325	1.246	4.153	71.325	2.756	9.186	71.325

Reliability and convergent validity were first examined. In PLS-SEM, standardized item loadings, Cronbach’s alpha, and composite reliability (CR) should generally exceed 0.70, while average variance extracted (AVE) should exceed 0.50 to ensure internal consistency and convergent validity (Fornell and Larcker, 1981; Hair et al., 2021). As shown in Table 6, all standardized factor loadings exceeded 0.70, ranging from 0.774 to 0.881. Cronbach’s alpha values ranged from 0.836 to 0.906, CR values ranged from 0.891 to 0.927, and all AVE values exceeded 0.50, indicating satisfactory reliability and convergent validity.

**Table 6:** Reliability and convergent validity.

Variables	Items	Factor loadings	Cronbach’s Alpha	CR	AVE
Event brand image recognition	BI1	0.872	0.887	0.922	0.747
	BI2	0.850			
	BI3	0.870			
	BI4	0.865			
Event emotional involvement	EI1	0.867	0.884	0.927	0.741
	EI2	0.881			
	EI3	0.856			
	EI4	0.840			
Landscape–cultural aesthetics	LA1	0.848	0.836	0.891	0.671
	LA2	0.835			
	LA3	0.795			
	LA4	0.797			
Order-and-care experience	OC1	0.834	0.851	0.906	0.692
	OC2	0.857			
	OC3	0.774			
	OC4	0.861			
Place bonding	PB1	0.842	0.906	0.927	0.681
	PB2	0.779			
	PB3	0.859			
	PB4	0.801			
	PB5	0.834			
	PB6	0.833			
Urban spatial meaning reconstruction	SR1	0.824	0.862	0.906	0.707
	SR2	0.841			
	SR3	0.835			
	SR4	0.863			
Scene transition perception	ST1	0.854	0.862	0.906	0.706
	ST2	0.848			
	ST3	0.842			
	ST4	0.817			

BI = event brand image recognition; EI = event emotional involvement; LA = landscape–cultural aesthetics; OC = order-and-care experience; PB = place bonding; SR = urban spatial meaning reconstruction; ST = scene transition perception.

Discriminant validity was further assessed using the Fornell–Larcker criterion and the heterotrait–monotrait ratio (HTMT) (Fornell and Larcker, 1981; Henseler et al., 2015). As presented in Table 7, the square root of the AVE for each latent variable was greater than its correlations with other latent variables, indicating adequate discriminant validity. Table 8 shows that all HTMT values were below 0.85, with a maximum value of 0.603, below the recommended threshold. Therefore, the discriminant validity of the measurement model was acceptable.

3 Structural model assessment

**Table 7:** Fornell–Larcker criterion.

Variables	BI	EI	LA	OC	PB	SR	ST
BI	**0.864**						
EI	0.436	**0.861**					
LA	0.343	0.503	**0.819**				
OC	0.373	0.519	0.330	**0.832**			
PB	0.340	0.494	0.481	0.412	**0.825**		
SR	0.423	0.528	0.420	0.497	0.525	**0.841**	
ST	0.390	0.465	0.398	0.453	0.470	0.487	**0.840**

Bold values on the diagonal represent the square roots of AVE.

**Table 8:** Heterotrait–monotrait ratio (HTMT).

Variables	BI	EI	LA	OC	PB	SR	ST
BI							
EI	0.491						
LA	0.397	0.581					
OC	0.428	0.596	0.385				
PB	0.380	0.552	0.546	0.468			
SR	0.480	0.603	0.491	0.579	0.590		
ST	0.441	0.529	0.464	0.524	0.528	0.559	

Before testing structural paths, model collinearity was assessed. VIF values should generally be below 3.3 or 5 to avoid serious multicollinearity. R2 assessed explanatory power, Q2 assessed predictive relevance, and f2 evaluated local effect size (Muller, 1989; Hair et al., 2021).

As shown in Table 9, all VIF values ranged from 1.274 to 1.788, below the commonly used threshold. This indicates that serious multicollinearity was not present.

**Table 9:** VIF values.

Path	VIF
BI - > EI	1.296
BI - > SR	1.347
EI - > PB	1.386
EI - > SR	1.788
LA - > EI	1.274
LA - > SR	1.422
OC - > EI	1.361
OC - > SR	1.513
SR - > PB	1.386
ST - > EI	1.441
ST - > SR	1.483

The model’s explanatory power and predictive relevance were then examined. As shown in Table 10, R2 values for event emotional involvement, urban spatial meaning reconstruction, and place bonding were 0.441, 0.418, and 0.341, respectively. Q2 values were 0.320, 0.288, and 0.229, all greater than 0, indicating adequate predictive relevance.

**Table 10:** *R*^2^ and *Q*^2^.

Variable	*R* ^2^	*Q* ^2^
EI	0.441	0.320
PB	0.341	0.229
SR	0.418	0.288

As shown in Table 11, the f^2^ effect sizes were generally small to nearly medium. Urban spatial meaning reconstruction had the largest effect on place bonding (*f*^2^ = 0.147), indicating that it was an important antecedent of place bonding. Landscape–cultural aesthetics had a relatively strong effect on event emotional involvement (*f*^2^ = 0.116), as did order-and-care experience (*f*^2^ = 0.112). These results suggest that landscape–cultural aesthetics and order-and-care experience are important in stimulating runners’ emotional involvement in the event.

4 Hypothesis testing

**Table 11:** *F*^2^ values.

Path	f2
BI - > EI	0.039
BI - > SR	0.024
EI - > PB	0.099
EI - > SR	0.041
LA - > EI	0.116
LA - > SR	0.018
OC - > EI	0.112
OC - > SR	0.051
SR - > PB	0.147
ST - > EI	0.029
ST - > SR	0.043

**Figure 5:**
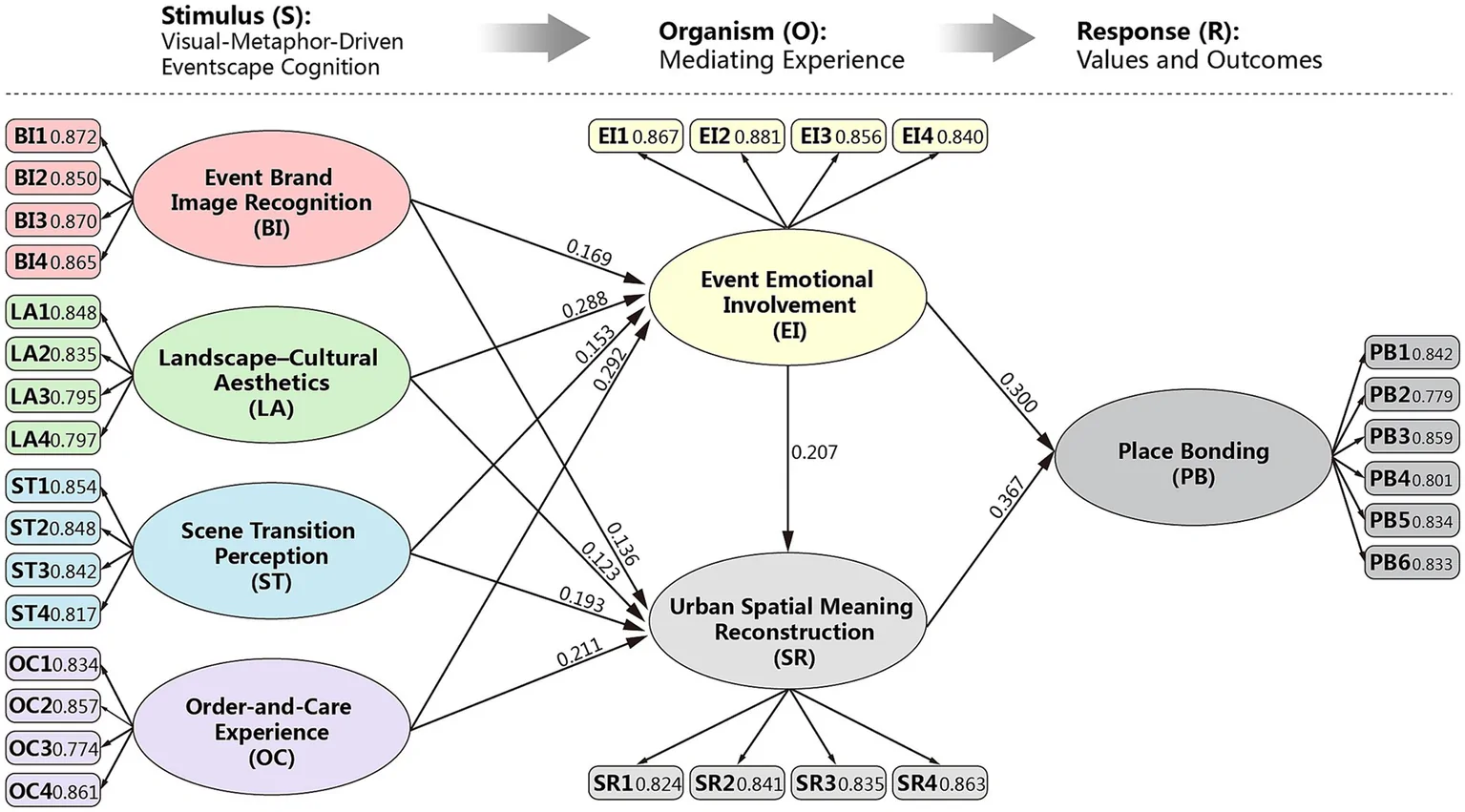
Structural model.

Bootstrap resampling was used to test the structural paths and mediation effects (Henseler et al., 2009; Hair et al., 2021). As shown in Figure 5 and Table 12, all direct paths were significant, supporting H1-H5. The four dimensions of visual-metaphor-driven eventscape cognition had significant positive effects on both event emotional involvement and urban spatial meaning reconstruction. Order-and-care experience had the strongest effect on event emotional involvement (*β* = 0.292, *p* < 0.001), followed by landscape–cultural aesthetics (*β* = 0.288, *p* < 0.001). For urban spatial meaning reconstruction, the effects of order-and-care experience (*β* = 0.211, *p* < 0.001) and scene transition perception (*β* = 0.193, *p* < 0.001) were more pronounced. These results indicate that visible social support, route-based landscape and cultural resources, and spatial transition are important sources of emotional involvement and the psychological reinterpretation of urban space.

**Table 12:** Path coefficient results.

Hypothesis	Paths	*β*	*T* statistics	*p*-values	Result
H1a	BI - > EI	0.168600224	3.563774926	0.000	Supported
H1b	LA - > EI	0.287975004	5.723033901	0.000	Supported
H1c	ST - > EI	0.1526142	2.685885134	0.007	Supported
H1d	OC - > EI	0.291747295	5.198188653	0.000	Supported
H2a	BI - > SR	0.13643028	2.676735678	0.007	Supported
H2b	LA - > SR	0.123219062	2.424743306	0.015	Supported
H2c	ST - > SR	0.192528451	3.592927774	0.000	Supported
H2d	OC - > SR	0.210993382	3.668471901	0.000	Supported
H3	EI - > SR	0.207273415	3.693139832	0.000	Supported
H4	EI - > PB	0.300468733	5.484845072	0.000	Supported
H5	SR - > PB	0.366695034	6.668833224	0.000	Supported

Event emotional involvement had a significant positive effect on urban spatial meaning reconstruction (*β* = 0.207, *p* < 0.001). Both event emotional involvement and urban spatial meaning reconstruction significantly and positively affected place bonding. Urban spatial meaning reconstruction had a stronger effect on place bonding (*β* = 0.367, *p* < 0.001) than event emotional involvement (*β* = 0.300, *p* < 0.001).

The mediation results are shown in Table 13. Event emotional involvement and urban spatial meaning reconstruction significantly mediated the relationships between the four dimensions of eventscape cognition and place bonding, supporting H6 and H7. The sequential mediation effects through event emotional involvement and urban spatial meaning reconstruction were also significant, supporting H8.

5 Robustness checks

**Table 13:** Mediation effect results.

Mediating path	Effect	*T* statistics	*P-*values	95% confidence interval	
				Lower bound	Upper bound
BI - > EI - > PB	0.051	3.117	0.002	0.021	0.085
LA - > EI - > PB	0.087	3.667	0.000	0.045	0.137
ST - > EI - > PB	0.046	2.190	0.029	0.011	0.092
OC - > EI - > PB	0.088	3.906	0.000	0.047	0.136
BI - > SR - > PB	0.050	2.627	0.009	0.013	0.089
LA - > SR - > PB	0.045	2.176	0.030	0.007	0.088
ST - > SR - > PB	0.071	2.852	0.004	0.029	0.125
OC - > SR - > PB	0.077	3.214	0.001	0.032	0.127
BI - > EI - > SR - > PB	0.013	2.225	0.026	0.004	0.026
LA - > EI - > SR - > PB	0.022	2.820	0.005	0.009	0.039
ST - > EI - > SR - > PB	0.012	1.992	0.046	0.002	0.025
OC - > EI - > SR - > PB	0.022	2.772	0.006	0.009	0.040

To examine the stability of the structural model estimates, three supplementary robustness checks were conducted. First, gender, age, education level, occupation, familiarity with Wuxi, number of Wuxi Marathon participations, and frequency of sport event participation in the past year were included in the model as control variables. Second, the measurement indicators were subjected to two-sided 1% winsorization, and the model was re-estimated to reduce the potential influence of extreme responses. Third, an alternative bootstrap specification with 1,000 subsamples was applied. As shown in Table 14, the signs of all path coefficients remained unchanged across the three specifications, the coefficient magnitudes varied only modestly, and all paths remained statistically significant at *p* < 0.05. These results provide additional evidence that the principal structural associations remain stable after controlling for demographic and participation-related characteristics, treating extreme values, and applying an alternative resampling specification.

**Table 14:** Results of supplementary robustness checks.

Paths	Model with control variables			Model with two-sided 1% winsorization			Alternative bootstrap estimation (1,000 subsamples)		
	*β*	*T* statistics	*P* values	*β*	*T* statistics	*P*-values	*β*	*T* statistics	*P*-values
BI - > EI	0.162	3.363	0.001	0.174	3.619	0.000	0.164	3.260	0.001
LA - > EI	0.288	5.800	0.000	0.268	5.180	0.000	0.277	5.057	0.000
ST - > EI	0.145	2.593	0.010	0.133	2.249	0.025	0.121	2.004	0.045
OC - > EI	0.299	5.327	0.000	0.296	5.176	0.000	0.296	4.691	0.000
BI - > SR	0.140	2.756	0.006	0.127	2.361	0.018	0.145	2.441	0.015
LA - > SR	0.122	2.403	0.016	0.124	2.400	0.016	0.134	2.578	0.010
ST - > SR	0.190	3.491	0.000	0.199	3.563	0.000	0.153	2.867	0.004
OC - > SR	0.200	3.402	0.001	0.195	3.347	0.001	0.244	3.981	0.000
EI - > SR	0.222	3.750	0.000	0.211	3.685	0.000	0.172	2.914	0.004
EI - > PB	0.293	5.299	0.000	0.254	4.700	0.000	0.242	4.175	0.000
SR - > PB	0.345	6.185	0.000	0.365	6.543	0.000	0.315	5.367	0.000

### 5.4 Discussion

The results of Study 2 indicate that visual metaphors in urban marathon participation operate through an integrated psychological mechanism. Brand, landscape, spatial transition, and order-and-care cues provide runners with continuous perceptual materials, which are processed through emotional involvement and the psychological reconstruction of spatial meaning. The path results highlight the central role of order-and-care experience. Together with landscape–cultural aesthetics, it strengthens emotional investment, ritual sense, and shared participation; together with scene transition perception, it supports the reorganization of urban encounters.

From the perspective of psychological processing, the visual metaphor system can be understood as a process of “perceptual input-internal processing-value linkage.” Visual-material elements are recognized, evaluated, and remembered by runners. Event emotional involvement and urban spatial meaning reconstruction then perform the internal meaning-conversion function, transforming immediate event participation into the reconnection, interpretation, and re-signification of urban space. The stronger effect of urban spatial meaning reconstruction on place bonding indicates that place bonding depends heavily on runners’ psychologically renewed understanding of the urban route and host city.

## 6 Conclusion

### 6.1 Main findings

Taking the 2025 Wuxi Marathon as a case, this study used A mixed-methods design to examine how visual metaphors shape runners’ place bonding in urban marathon participation. The study first used metaphor elicitation to identify runners’ meaning chains of visual perception and then used PLS-SEM to validate the key structural relationships. The findings show that place bonding in urban marathons emerges from the coordination of event visual symbols, urban spatial encounters, bodily movement, and social interaction. This configuration echoes systemic accounts of urban visuality and previous research connecting visual design with affective place branding (Aiello, 2021b; Zhang et al., 2025).

First, Study 1 revealed the consensus structure of visual metaphors in this case. Across pre-race, in-race, and post-race encounters, runners’ highly perceived cues included the event visual identity system, the scenic landscape-waterscape route, landmark and cultural displays, youthful campus scenes, and the order-and-care system. These cues formed four pathways of meaning generation: brand symbolization, embodied landscape–cultural experience, urban spatial reorganization, and socio-emotional bonding. Related research on event experience, eventscape, and destination sensescape has likewise highlighted the functional, environmental, social, and affective dimensions of participation (Carneiro et al., 2019; Girish and Lee, 2019; Buzova et al., 2021; Chen and Shi, 2025). In the present case, these dimensions were organized as visual-metaphorical cues through which runners generated place meaning.

Second, Study 2 validated the quantitative mechanism of visual metaphors. The results indicate that visual metaphor cues are associated with stronger place bonding through the sequential mediating effects of emotional involvement and urban spatial meaning reconstruction. Comparable associations between cultural and environmental atmospheres, positive affect, and event-related responses have been reported in previous studies (Song et al., 2025; Wang et al., 2025). The present results show that these relationships operated through the combined mediation of event emotional involvement and urban spatial meaning reconstruction.

Third, the psychological reconstruction of urban spatial meaning is the key mediating mechanism through which visual metaphors are transformed into place bonding. The place value of urban marathons is shaped by event-related emotional responses and, more strongly, by runners’ reconstruction of mental maps and renewed understanding of urban space during movement. This finding parallels evidence that bodily behavior and movement shape participants’ evaluations of both the city and the event (Xu and Dai, 2025). In this case, route-based spatial linking was central to runners’ renewed place cognition.

Fourth, order-and-care experience plays a pivotal role within the visual metaphor mechanism. Existing empirical findings support the view that visible service cues can stimulate runners’ emotional involvement and shape their overall judgment of a city’s friendliness, organizational capacity, and affective warmth (Lee et al., 2018; Lin et al., 2024). Such city support, when embedded in the environmental system of the event, can also respond to participants’ needs for a better life (Ma and Huang, 2026). The participation of volunteers, spectators, and supporters in sporting events can also enhance affective recognition of both the event and the city (Song et al., 2025). The two phases in this study provide complementary evidence: ZMET primarily linked order-and-care experience to emotional support and shared participation, while PLS-SEM showed that it also contributed to urban spatial meaning reconstruction.

Overall, this study conceptualizes urban marathon participation as a visual-metaphor-driven process of place meaning generation. Place bonding is generated through a continuous process that links visual metaphor cues, eventscape cognition, emotional involvement, and the psychological reconstruction of spatial meaning. The findings show that short-term, high-intensity, and mobile sport events can provide a meaningful setting for the formation of place bonding.

### 6.2 Theoretical contributions

This study extends the explanatory perspective of urban marathon research by foregrounding runners’ psychological experience. The findings advance existing research in three respects.

First, existing eventscape research has established the influence of physical and social event environments on participant attachment and loyalty, while mobility research has highlighted the role of movement in people–place relationships (Zhang, 2022; Kołodyńska et al., 2025). Building on these conclusions, this study advances eventscape research from examining separate attribute effects to explaining how event and urban environments are experientially integrated. The visual-metaphor-driven eventscape cognition framework provides a relational account of how distributed visual, spatial, and social cues are organized around the host city and converted into place meaning.

Second, research on place bonding and place attachment has conceptualized person–place relationships as multidimensional and developing through meaningful interactions among people, activities, and settings (Hammitt et al., 2006; Cheng and Kuo, 2015; Han et al., 2019). Research on event-based recreation further suggests that such relationships may emerge through event participation, while place experience involves embodied, psychological, and socially mediated spatial processes (Buttazzoni et al., 2022; Budruk et al., 2024). The present study advances this knowledge by clarifying the sequential relationship and relative roles of affective and interpretive processes in place bonding. The significant sequential mediation supports a process in which event emotional involvement contributes to urban spatial meaning reconstruction, while the stronger effect of the latter identifies urban interpretation as the more immediate basis of place bonding. This refines existing accounts that primarily treat affective and cognitive outcomes as parallel relationships.

Third, expanded servicescape theory has already moved beyond the physical service setting by incorporating social, socially symbolic, and natural environmental dimensions (Rosenbaum and Massiah, 2011). The findings in this study extend this perspective from service and event evaluation to place-level cognition. By linking order-and-care experience with urban spatial meaning reconstruction, the study reframes visible service environments as resources through which the wider host setting is psychologically interpreted. This indicates that event services participate in the construction of place meaning as well as event delivery.

Beyond these theoretical contributions, the study also offers a methodological contribution. The integration of ZMET and PLS-SEM connects participant-generated meanings with structural mechanism testing, allowing context-specific experiential constructs to enter quantitative model development. It thereby provides a context-grounded complement to research based primarily on predefined constructs and established scales.

### 6.3 Practical implications

Based on the finding that visual metaphors operate as an integrated system of place meaning generation, this study proposes four interrelated strategies for developing urban experience-oriented marathons into sustainable platforms for event–city value creation. These strategies can be adapted to different cultural, spatial, and organizational contexts.

First, the qualitative findings concerning event identity and participatory meaning support the development of a shared event–city visual system. Events can establish a stable cultural motif across annual editions and build a participatory visual asset library using images, stories, and design proposals contributed by residents and runners. Selected event graphics or commemorative installations can also remain in public spaces after the race, allowing event-generated visual value to accumulate over time.

Second, the stronger role of urban spatial meaning reconstruction indicates that the route should be planned as an integrated urban experience. Routes can connect landmarks with universities, historic neighborhoods, industrial heritage, everyday communities, and other locally meaningful spaces. At major scene-transition points, organizers can use flush-mounted pavement graphics, concise place-based story panels, and distinctive route markers that remain legible at running speed. These devices can help runners connect individual scenes with the wider spatial and historical structure of the city.

Third, the prominent role of order-and-care experience suggests that service provision should function as visible and adaptive care infrastructure. Service stations, guidance systems, volunteer support, and cheering areas can share a coherent visual language and incorporate locally meaningful content. Hierarchical wayfinding, accessible and multilingual information, differentiated supply arrangements, medical support, and real-time emergency response can address differences in runners’ abilities, experience, and familiarity with the city. Crowd-density and service-availability data may also support timely adjustments to guidance and supplies.

Fourth, the sequential relationship among eventscape cognition, emotional involvement, spatial meaning reconstruction, and place bonding supports lifecycle-based event–city management. Pre-event visual exploration guides and route-preview activities can build initial familiarity with the host environment. During the race, the official event app can automatically associate key route segments with relevant local stories and save them for post-race review, without requiring active interaction while running. After the event, personalized route archives, runner communities, and city revisit programs can preserve and reactivate event meanings. With participant consent, personalized content recommendations may further connect route memories with related urban spaces and future cultural activities.

Together, these strategies integrate visual communication, route design, service provision, and continuing engagement within a coordinated event–city system. Technology provides selective support for their implementation. By coordinating physical environments, social care, and digital mediation, these practices may also create conditions that support psychological well-being through positive emotional experience, perceived care and support, and meaningful relationships with urban places.

### 6.4 Limitations and future research

This study has several limitations. First, it uses the 2025 Wuxi Marathon as a single case. Its cherry blossom seasonality, scenic landscape-waterscape route resources, and mature event brand system are strongly embedded in the local context. The findings therefore need to be further tested in other urban events. Second, Study 1 relied mainly on post-event recall and participant-selected images, which limited the capture of real-time bodily perception and emotional changes during the race. Third, Study 2 used cross-sectional questionnaire data, and the long-term development and causal direction of place bonding require further longitudinal examination. Fourth, the supplementary robustness checks focused on the inclusion of control variables, extreme-value treatment, and alternative resampling specifications. Although these analyses support the stability of the principal path estimates across the tested specifications, they were not designed to provide a dedicated assessment of latent heterogeneity or potential endogeneity.

Future research can proceed in four directions: multi-case comparison, dynamic data collection, longitudinal validation, and more extensive model robustness assessment. Subsequent studies may compare marathons hosted by cities with different characteristics, such as historical-cultural, coastal, mountain, or emerging-city events, to examine the applicability of the visual metaphor mechanism. Researchers may also combine mobile interviews, experience sampling, route observation, movement trajectories, or physiological data to capture the dynamic process of runners’ experience. In addition, data could be collected before the event, immediately after the event, and several months later to examine temporal changes in visual metaphor perception, the psychological reconstruction of urban spatial meaning, and place bonding. Research based on larger and more heterogeneous samples may further apply latent-class and complementary endogeneity diagnostics to examine the consistency of the structural relationships across unobserved subgroups and alternative model specifications.

In conclusion, this study understands urban marathon participation as a temporary embodied experience capable of generating place meaning. Its value lies in fostering durable spatial cognition and place-based psychological relationships through visual perception, bodily experience, affective memory, and spatial understanding. Future research may continue to examine how such event-based experiences participate in the generation, continuation, and reproduction of urban meaning, as well as their long-term role in enhancing urban affective resilience, public participation, and social inclusiveness.

## Data Availability

The original contributions presented in the study are included in the article. Further inquiries can be directed to the corresponding author.
